# Topical Fibrin Sealant (Tisseel@) Does Not Provide a Synergic Blood-Conservation Effect with Tranexamic Acid in Total Knee Arthroplasty—A Prospective Randomized Controlled Trial

**DOI:** 10.3390/medicina59122078

**Published:** 2023-11-26

**Authors:** Chia-Hung Liu, Chih-Hsiang Chang, Yu-Han Chang, Hsin-Nung Shih, Chih-Chien Hu

**Affiliations:** 1 Department of Orthopedic Surgery, Chang Gung Memorial Hospital, Taoyuan 333, Taiwan; liu.hong.8187@gmail.com; 2 Division of Joint Reconstruction, Department of Orthopedic Surgery, Chang Gung Memorial Hospital, Taoyuan 333, Taiwan; 8802032@cgmh.org.tw (C.-H.C.); yhchang@cgmh.org.tw (Y.-H.C.); aronc@cgmh.org.tw (H.-N.S.)

**Keywords:** Tisseel@, tranexamic acid (TXA), total knee arthroplasty (TKA), blood loss

## Abstract

*Background and Objectives*: The efficacy of tranexamic acid (TXA) in reducing perioperative blood loss during total knee arthroplasty (TKA) is well established. However, the potential synergistic blood-conservation effect of topical fibrin sealant (Tisseel@) remains unclear. This study aims to assess the effectiveness of the combination of Tisseel and TXA during TKA. *Materials and Methods*: A single-blinded, prospective, randomized controlled trial was conducted with 100 patients (100 knees) undergoing primary TKA. Participants were randomly assigned to either the TXA group (n = 50), receiving intravenous (IV) TXA, or the Tisseel@ + TXA group (n = 50), receiving intra-articular Tisseel@ combined with IV TXA. The primary outcomes included blood transfusion rate, decrease in Hb level, calculated blood loss, and estimated total postoperative blood loss. Secondary outcomes involved assessing clinical differences between the groups. *Results*: The transfusion rate was zero in both groups. The average estimated blood loss in the Tisseel@ + TXA group was 0.463 ± 0.2422 L, which was similar to that of the TXA group at 0.455 ± 0.2522 L. The total calculated blood loss in the Tisseel@ + TXA group was 0.259 ± 0.1 L, compared with the TXA group’s 0.268 ± 0.108 L. The mean hemoglobin reduction in the first 24 h postoperatively was 1.57 ± 0.83 g/dL for the Tisseel@ + TXA group and 1.46 ± 0.82 g/dL for the TXA-only group. The reduction in blood loss in the topical Tisseel@ + TXA group was not significantly different from that achieved in the TXA-only group. The clinical results of TKA up to the 6-week follow-up were comparable between the groups. *Conclusions*: The combination of the topical fibrin sealant Tisseel@ and perioperative IV TXA administration, following the described protocol, demonstrated no significant synergistic blood-conservation effect in patients undergoing TKR.

## 1. Introduction

The concept of enhanced recovery after surgery (ERAS) was introduced in the 1990s by the Danish surgeon H. Kehlet [1]. Kehlet advocated a series of measures to optimize perioperative care, aiming to reduce both physical and psychological trauma and stress after surgery, consequently expediting the recovery process. The fundamental principle of ERAS is centered around minimizing postoperative complications and costs, shortening the length of hospital stay (LOS), enhancing patient satisfaction, and promoting faster recovery. One of the key elements is the reduction in blood loss during and after the surgical procedure [2,3,4]. Total knee arthroplasty (TKA) is a widely accepted and effective treatment for end-stage arthritis of the knee. However, it has traditionally been associated with considerable blood loss and subsequent complications. Studies have reported post-TKA blood loss ranging from 1400 to 1800 mL, with blood transfusion rates reaching as high as 40% [5]. Increased postoperative bleeding may result in increased pain, hematoma, poor wound healing, surgical-site infection, postoperative anemia, and associated complications. According to active members of the American Association of Hip and Knee Surgeons (AAHKS) regarding their current preferences and practices, the average transfusion rate after undergoing unilateral knee replacement was estimated to be less than 5%, with a range spanning from 0% to 20%. For patients who underwent bilateral knee replacement, the estimated transfusion rate fell between 10% and 20%, with a range of 5% to 20% [6]. Therefore, achieving ERAS certainly involves minimizing perioperative bleeding.

Tranexamic acid (TXA), a fibrinolysis inhibitor, has demonstrated efficacy in blood conservation following TKA when administered either intravenously or topically to the knee joint during the surgical procedure [7,8,9]. According to our previous report, the application of topical tranexamic acid in patients undergoing total hip arthroplasty effectively reduces postoperative bleeding and leads to a decrease in blood transfusion rates [10]. Therefore, the routine protocol in our clinical arthroplasty application involved the use of either topical or IV TXA, following the recommendations of Enrique et al. [11]. Since 1972, the adjunctive use of fibrin sealants in certain surgical procedures has been adopted as an optional approach to manage hemostasis and minimize blood loss after surgical interventions. Nevertheless, the efficacy of fibrin sealants in TKA surgery has not yet attained the status of a standard practice [12]. Review articles and meta-analysis reports have indicated that high-dosage fibrin sealant is effective and safe as a hemostatic therapy for patients undergoing TKA [13,14,15]. However, there are limited reports comparing the hemostatic ability of fibrin sealants and TXA or the combined usage of both regimens [16]. We are interested in exploring whether the application of the fibrin sealants Tisseel@ and TXA to the knee joint may lead to a synergistic effect in reducing perioperative blood loss during TKA. If successful, the primary TKA procedure will tend to align with the goals of ERAS. Therefore, the current study aims to determine whether an isolated IV TXA injection protocol or a combination Tisseel@ + IV TXA protocol results in reduced blood loss in TKA patients. Additionally, we aim to investigate whether perioperative complications and postoperative recovery differ with the combination of Tisseel@ and TXA during surgery. We hypothesize that the addition of topical fibrin sealant (Tisseel^®^) can provide a synergistic blood-conservation effect with TXA in patients undergoing TKA.

## 2. Materials and Methods

### 2.1. Study Design

This prospective randomized controlled trial protocol was registered with ClinicalTrials.gov under the registration number NCT03310060. Additionally, it received approval from the institutional review board and support from Chang Gung Memorial Hospital (IRB no. 201700271A3). Before surgery, informed consent was obtained from all participants. The manuscript was prepared following the Consolidated Standards of Reporting Trials (CONSORT) guidelines (as detailed in Table S1 of the Supplementary Materials) [17].

### 2.2. Inclusion and Exclusion Criteria

Between September 2017 and August 2019, we consecutively assessed a series of 119 patients who underwent unilateral primary TKA for initial eligibility in this study. The inclusion criteria encompassed adult patients aged 20 to 80 years with end-stage arthritis of the knee joint necessitating TKA. The exclusion criteria for this study were as follows: patients with non-OA degenerative knee joints (e.g., rheumatoid arthritis or septic arthritis), patients with severe anemia (hemoglobin/hematocrit (Hb/Hct) < 12 g/dL/ < 36% before surgery), patients with end-stage renal failure undergoing dialysis treatment, liver cirrhosis leading to abnormal coagulation function (Child A/B/C conditions, platelets < 100,000/µL), patients using any oral anticoagulants or antiplatelets, patients with abnormal coagulation function (INR > 1.5 s), body mass index (BMI) exceeding the standard (35 < BMI < 18), a history of anaphylactic shock or severe allergic reaction to bovine protein (aprotinin), and women who were pregnant and/or breastfeeding.

### 2.3. Intervention Protocol

The patients were randomly assigned to two groups: the TXA group and the Tisseel@ + TXA group. All TKAs were performed by the same experienced surgical team (H.N.S., Y.H.C, and C.C.H.) using the mini-midvastus approach described by Haas et al., under general anesthesia [18]. Before the surgical incision, a pneumatic tourniquet was applied and inflated to a pressure 100 mmHg higher than the patient’s systolic blood pressure. This tourniquet was kept in place during the surgery and deflated at the end of the procedure. To control bleeding during the surgery, routine hemostasis was accomplished by electrocoagulation of bleeding points. All TKAs used the same prosthesis (U2 Knee Posterior-Stabilized Prosthesis; United^®^, Hsinchu City, Taiwan). Femoral cutting was guided using intramedullary guidance, and an extramedullary guidance system was employed for the tibial site. After adequate implantation of the prosthesis with a fully cementing technique, a Hemovac (intra-articular drainage tube) was placed. The total volume of drained blood was monitored and recorded every 8 h during the postoperative period.

In the TXA group, patients received an intravenous injection of 15 mg/kg of TXA (Transamin 100 mg/mL; China Chemical and Pharmaceutical Co., Taipei City, Taiwan) before the surgical incision in the operating theater. An additional dose of IV TXA was administered 3 h after the surgery. Patients in the Tisseel@ + TXA group received the same IV TXA protocol before and after surgery. Concurrently with the surgery, a 4 mL Tisseel@ (Baxter^®^ AG, Vienna, Austria) intra-articular spray was applied to the raw surfaces of bone resection after femoral and tibial cutting. Additionally, it was used on bleeding points of the soft tissue and the pinhole before the closure of the arthrotomy. As part of the routine procedure, manual compression of the Tisseel fibrin glue onto the bone surface or soft tissue using wet gauze was performed for a duration of 2 min. The Hemovac drain was routinely clamped for 6 h and then released for open drainage, before being removed 24 h after surgery. The amount of crystalloid solutions was not recorded. At the conclusion of surgery, hemoglobin (Hb) and hematocrit (Hct) levels were measured. On the first day after the surgery, Hb and Hct were reevaluated. IV prophylactic antibiotic treatment was administered, which included 1 g of cefazolin before the operation, followed by 1 g every 8 h for three doses after the surgery. Standard thromboembolic prophylaxis was prescribed to all patients, involving oral intake of 100 mg of aspirin once daily, starting on the day after the operation, and continued for 14 doses.

### 2.4. Outcomes

Originally, the protocol for the study designated only the total blood loss as the primary outcome. However, after team discussions, it was deemed meaningful to explore potential differences between the two groups in terms of blood transfusion rate, decrease in Hb level, and calculated blood loss. Consequently, we decided to include blood transfusion rate, decrease in Hb level, calculated blood loss, and invisibly estimated total blood loss as primary outcomes in our study design. Allogeneic blood transfusion of red blood cells was triggered when the hemoglobin (Hb) level dropped below 8 g/dL, or when any signs of hypovolemic shock were observed. We calculated the patient’s blood volume (PBV) based on Nadler’s formula [19]. The estimated blood loss was calculated using the formula described by Gross, which is primarily based on changes in Hct values [20] (as detailed in Table S2 of the Supplementary Materials). Calculated blood loss was the combination of intraoperative blood loss according to suction and gauge combined with the Hemovac record. The secondary outcomes included functional recovery, pain control assessed with visual analogue scales [21], length of hospital stay, and the rate of complications, including wound condition, hematoma, and superficial skin necrosis, in addition to deep-vein thrombosis or potential periprosthetic joint infection [22]. All patients received follow-up care at the orthopedic clinic, with evaluations conducted at 2 weeks and 6 weeks post-surgery, and up to 2 years thereafter. Pain levels, symptoms, activities of daily living, sports and recreation function, and knee-related quality-of-life outcomes were recorded at 2 weeks and 6 weeks after the operation, utilizing the Knee Injury and Osteoarthritis Outcome Score (KOOS) scoring system [23].

To further assess the practical significance of the observed differences between the TXA group and the Tisseel@ + TXA group, we calculated Cohen’s d as a measure of effect size. This effect size will provide additional insights into the magnitude of the observed differences in blood loss between the two treatment groups.

### 2.5. Randomization and Blinding

The randomization was conducted by an independent research assistant using a computer-generated method, based on the sequence of operation dates. The clinical investigators remained blind to the randomization and allocation of all patients until complete data had been collected.

### 2.6. Minimum Sample Size

The determination of the sample size was based on the research conducted by Molloy et al., who conducted a prospective randomized trial to assess perioperative blood loss following TKA [16]. To demonstrate a difference of 0.5 g/dL in the postoperative decrease in hemoglobin (Hb) levels, with a significance level (α) of 0.05, a power of 90% (1–β), and a β-value of 0.10, it was determined that 50 patients would be required in each group.

### 2.7. Statistical Analysis

Statistical analyses were performed using the Statistical Package for Social Sciences (SPSS) software (Version 22.0; SPSS Inc., Chicago, IL, USA). The analysis of continuous variables utilized Student’s *t*-test, while the chi-squared test was employed for dichotomous variables. A *p*-value less than 0.05 was considered to be statistically significant, indicating a meaningful result. The effect size of the calculated blood loss and estimated total blood loss was determined using Cohen’s d. Cohen’s d is calculated by subtracting the means of two groups and dividing the result by the pooled standard deviation. The interpretation of Cohen’s d is as follows: a small effect is indicated when d is approximately 0.2, a medium effect when d is approximately 0.5, and a large effect when d is approximately 0.8 [24].

## 3. Results

### 3.1. Participants’ Flow

**Figure 1:**
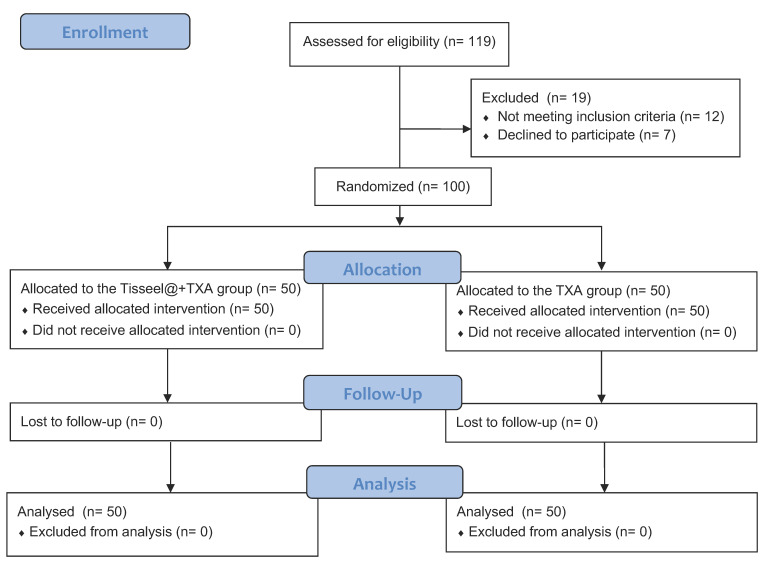
CONSORT 2010 participant flow diagram.

The participant flow is illustrated in Figure 1. Initially, 119 patients were enrolled, but 12 of them were excluded due to meeting the exclusion criteria during screening. Most of these exclusions were related to anemia (Hb < 12.0 g/dL) or long-term administration of oral anticoagulants or antiplatelets. Additionally, seven patients were excluded because they did not undergo surgery due to administrative issues. Thus, a total of 100 patients were included in this study. The patients were randomly allocated into two categories: the TXA group and the Tisseel@ + TXA group. Ultimately, complete data for analysis were available for 50 patients in the TXA group and 50 patients in the Tisseel@ + TXA group.

### 3.2. Baseline Data

The preoperative demographics of the patients, including age, gender, body mass index (BMI), preoperative hemoglobin (Hb) level, hematocrit (Hct), international normalized ratio (INR), platelet count, and American Society of Anesthesiologists (ASA) grade, underwent comparative analysis between the two groups (Table 1). The only significant difference identified between the groups was in gender; however, the allocation was based on a randomly assigned sequence, without manipulation.

### 3.3. Primary and Secondary Outcomes

Among the participants, none required blood transfusions perioperatively, resulting in a transfusion rate of zero in both groups. In the Tisseel@ + TXA group, the average estimated blood loss was 0.463 ± 0.2422 L, which was comparable to the TXA group’s value of 0.455 ± 0.2522 L (*p* = 0.9). Additionally, there were no significant differences observed in the decrease in Hb levels on post-OP day 1 between the Tisseel@ + TXA group (1.57 ± 0.83 g/dL) and the TXA group (1.46 ± 0.82 g/dL). The postoperative drainage volume was similar in both groups, with values of 0.148 ± 0.099 L in the Tisseel@ + TXA group and 0.155 ± 0.099 L in the TXA group. Consequently, the calculated total blood loss was 0.259 ± 0.1 L in the Tisseel@ + TXA group and 0.268 ± 0.108 L in the TXA group (*p* = 0.341), indicating no significant differences between the two groups. Furthermore, the visual analogue scales, range-of-motion arc of the index knee, and length of hospital stay were similar in both groups. After 2 and 6 weeks following the surgery, there were no significant differences observed in the Knee Injury and Osteoarthritis Outcome Score (KOOS) between the groups (Table 2).

The assessment of perioperative wound conditions, from immediately after the surgery to six weeks post-operation, included evaluating ecchymosis, swelling caused by hematoma, wound-healing issues, and isolated occurrences of periprosthetic joint infection. The incidence of these conditions was found to be comparable between the studied groups. No patient in either group required a return to the operating theater due to any potential complications until two years postoperatively. A single patient experienced calf swelling with minimal pain at the 2-week postoperative follow-up, suspected to be deep-vein thrombosis. However, the patient declined aggressive management, even after duplex ultrasonography confirmation. Fortunately, the symptoms and signs had significantly improved at the 6-week follow-up, with no further complications (Table 3).

The effect size, as indicated by Cohen’s d, for the estimated total blood loss was 0.032382, while the effect size for calculated blood was 0.094433. Both of these findings suggest a small effect (Table 4).

## 4. Discussion

Making every effort to improve the reduction in blood loss during and after surgical procedures is a fundamental aspect of all surgeries. According to Gao et al., IV TXA has shown a potential association with reduced blood transfusion requirements and higher hemoglobin levels in patients undergoing TKA. Importantly, this benefit was observed without an increase in the incidence of postoperative complications compared to the use of topically applied fibrin sealants. Moreover, there was no significant difference noted in the total calculated blood loss between the two groups [25]. However, we wondered whether there was any possibility of a synergistic effect between these two methodologies, thus prompting the current study.

The findings from our prospective randomized controlled trial indicated that the incidence of blood transfusion was zero in both the combined Tisseel@ + IV TXA group and the IV TXA-only group. In the Tisseel@ + TXA group, the average estimated blood loss was 0.463 ± 0.2422 L, which was comparable to the TXA group’s value of 0.455 ± 0.2522 L. Additionally, postoperative calculated blood loss was comparable between the two groups, with values of 0.259 ± 0.1 L and 0.268 ± 0.108 L, respectively. These results suggest that no synergistic effect was observed when adding fibrin sealants (Tisseel@) to IV TXA in patients undergoing TKA. The absence of statistically significant differences in estimated total blood loss and calculated total blood loss between the two groups may be further clarified by examining Cohen’s d. The effect size is negligible and reinforces the idea that the addition of Tisseel@ may not provide a meaningful advantage over IV TXA in the context of blood conservation during TKA.

The primary objective of achieving ERAS for TKA is to implement a multimodal approach that minimizes surgical-related trauma and stress. This approach involves employing the least invasive surgical techniques and practices. Nevertheless, the reduction in blood loss during and after surgical procedures is one of the key elements of ERAS. Proper intraoperative management significantly contributes to ensuring safety, minimizing blood loss, and promoting early recovery. In the American College of Surgeons’ National Surgical Quality Improvement Program database, bleeding requiring transfusion was the most common complication in TKA patients who failed the ERAS protocol [3,26]. Therefore, perioperative anemia detection and management, monitoring anticoagulant/aggregant usage, transfusion threshold determination, anesthetic technique selection, local infiltration analgesia administration, drainage clamping and removal procedures, and intraoperative multimodal hemostasis are crucial factors that can greatly enhance the effectiveness of a fast-track pathway. These elements offer substantial opportunities for strategic planning and improved clinical outcomes [27].

TXA is a synthetic anti-fibrinolytic medication that acts as a competitive inhibitor, specifically blocking the lysine-binding sites of plasminogen, plasmin, and tissue plasminogen activator. By doing so, TXA effectively retards the process of fibrinolysis and prevents the breakdown of blood clots [28]. Regarding the IV administration of TXA in TKA patients, it has been documented to effectively diminish blood loss while maintaining a high level of safety [29]. The administration of TXA has found extensive application in various medical contexts, resulting in a decreased requirement for blood transfusions in surgeries such as cardiac, orthopedic, cranial, orthognathic, hepatic, and urological procedures [30,31]. Nonetheless, the method of TXA administration can vary, with options including intravenous administration or topical application directly to the surgical wound, and surgeons may have their own preferred approaches. Studies have demonstrated that intravenous TXA administration is both effective and safe in reducing blood loss and the need for blood transfusions during TKA when compared to control groups [32]. Consequently, the meta-analysis has reached a conclusion indicating that both IV and intraarticular administration of TXA for patients undergoing TKA are effective and safe in reducing blood loss and the need for blood transfusions. Moreover, this treatment approach does not increase the risk of postoperative deep-vein thrombosis [9,33].

The utilization of fibrin sealants, which are created by mixing plasma fibrinogen with thrombin, was initially reported during World War II [34]. Fibrin sealants employed as topical agents are composed of a combination of plasma fibrinogen and thrombin, resulting in the formation of a fibrin clot adhesive that effectively achieves hemostasis and stops bleeding. Numerous studies have extensively investigated fibrin sealants, and fibrin tissue adhesives are now utilized in a wide range of surgical disciplines. They find application in procedures like prostatectomy, partial pulmonary excision, carotid endarterectomy, and hepatectomy involving liver mobilization [35,36,37]. Yasukawa et al. reported that the combination of a fibrin sealant and intra-articular TXA administered as a ‘cocktail’ acted as hemostatic agents. As a result, the observed reduction in blood loss could not be solely attributed to the fibrin sealant alone [38]. Another study demonstrated that the application of the human fibrin sealant Quixil in patients undergoing TKA showed promising results. It potentially led to reduced blood loss and decreased transfusion rates, without any notable increase in adverse events [39]. A meta-analysis of the available literature demonstrated that the application of fibrin sealant in patients undergoing TKA resulted in reductions in total blood loss, drainage loss, calculated total hemoglobin loss, and transfusion rate [13]. However, no significant differences were observed in the incidence of DVT, surgical-related infections, ecchymosis, and hematoma between the fibrin sealant group and the control group. Consequently, based on the current meta-analysis, it appears that the use of fibrin sealants can be considered both effective and safe for patients undergoing TKA.

In the current study, only a single dosage of the fibrin sealant Tisseel@ was used in our trial. According to a meta-analysis published by Yang et al., the dosages in nine randomized control trials and four prospective comparative studies ranged from 2 mL to 10 mL of fibrin sealant. Their conclusion was that the utilization of fibrin sealant may lead to a reduction in the transfusion rate and the number of transfusion units required after TKA. However, the impact in terms of reducing the total blood loss was not significant [14]. The results of subgroup analyses showed consistent outcomes for both low dosages (2 mL) and high dosages (5~10 mL) of fibrin sealant. Even with the higher dosage of fibrin sealant, it did not prove to be effective in managing postoperative bleeding, improving functional recovery, or reducing complications. Wang et al. reported in their meta-analysis of fibrin sealants in TKA that the amount of fibrin sealant did not appear to have any bearing on the results [15]. Therefore, based on the available evidence, the efficacy of dose-dependent fibrin sealant in TKA surgery is inconclusive. This is the major reason why we chose 4 mL of the fibrin sealant Tisseel@ to conduct the current study, as this was the most cost-effective selection (4 mL and 8 mL dosage packages) in our market. Nevertheless, it is undeniable that a higher dose of fibrin sealant may provide a better hemostatic effect; however, the potential bleeding points, such as bone cuts and soft-tissue raw surfaces, were mostly well covered by the 4 mL of Tisseel@.

Our study has several limitations. First, the sample size in each group was relatively small. Nevertheless, in numerous prospective randomized controlled trials examining the variance in postoperative blood loss after TKA between groups, significant findings have been observed with fewer than 50 patients in each group [40,41]. Second, there were not enough control groups in this study, such as an independent fibrin sealant group, or a true negative control without any pharmaceutical intervention, to identify the influences of each independent factor. However, the design of the current study was to discover the synergistic effect of the fibrin sealant Tisseel@ on TXA, which may improve our routine TXA administration in clinical practice. Third, it should be noted that the fibrin sealants utilized in this study were limited to a single 4 mL dosage. However, since the fibrin sealants served as an adjunctive agent for sealing bleeding surfaces of the bone cuts and raw areas, the current dosage was found to be adequate for the intended purpose. Fourth, we did not conduct a small-scale pilot study before formally initiating the trial to determine the required sample size. Instead, an alternative approach was adopted by reviewing relevant literature and similar prior studies. The sample size for this trial was determined by referencing literature with comparable research objectives and methodologies, which may have introduced some flaws in the experimental design. In the end, even though the allocation was carried out according to a randomly assigned sequence without manipulation, a significant difference in gender distribution still emerged between the two groups. This outcome introduces a certain flaw in the judgment of comparability between the two groups.

## 5. Conclusions

Our prospective randomized controlled study did not demonstrate a synergistic blood-conservation effect when combining the use of Tisseel@ and TXA in patients undergoing unilateral primary TKA.

## Figures and Tables

**Table 1:** Demographic data.

	TXA (n = 50)	Tisseel@ + TXA (n = 50)	*p*-Value
Age (years)	68.48 (49~80)	71.42 (56~80)	0.3
Gender (female: male)	41:9	30:20	0.027
Body mass index (kg/m^2^)	28.75	27.22	0.224
Preoperative Hb (g/dL)	13.78 ± 1.2 (12.0~17.1)	13.67 ± 1.0 (12.2~16.4)	0.254
Preoperative Hct (%)	41.67	41.18	0.191
Preoperative INR	1.4	1.3	
Platelet count (1000/µL)	158	147	
American Society of Anesthesiologists (ASA) grade	2.8	2.7	

**Table 2:** Primary and secondary outcomes in both groups.

	TXA (n = 50)	Tisseel@ + TXA (n = 50)	*p*-Value
Blood transfusion (no./total no)	0/50	0/50	1
Hb level change on post-OP day 1	1.46 ± 0.82 (−0.2~3.4)	1.57 ± 0.83 (0.2~3.2)	0.892
Hct level change on post-OP day 1	4.92 ± 2.22 (0.9~10.1)	4.92 ± 2.33 (0.6~9.6)	0.498
Estimated total blood loss (L)	0.455 ± 0.2522 (0.083~1.213)	0.463 ± 0.2422 (0.057~1.074)	0.9
Drainage volume (L)	0.155 ± 0.099 (0~0.46)	0.148 ± 0.099 (0~0.42)	0.862
Intra-operative blood loss (L)	0.118 ± 0.051 (0.05~0.3)	0.111 ± 0.040 (0.05~0.2)	0.285
Calculated blood loss (L)	0.268 ± 0.108 (0.1~0.56)	0.259 ± 0.1 (0.55~0.52)	0.341
VAS post-OP day 1	2.3 (0~6)	2.6 (0~8)	
VAS post-OP day 2	2.1 (0~6)	2.3 (1~6)	
Length of hospital stay (days)	4.6	4.3	
Range-of-motion (ROM) arc at discharge day	116.8°	114.9°	
KOOS score (post-OP 2 weeks)	76.8	74.5	
KOOS score (post-OP 6 weeks)	86.5	86.9	

**Table 3:** Wound condition and perioperative complications.

	TXA (n = 50)	Tisseel@ + TXA (n = 50)
Wound length in extension (cm)	8.6	9.1
Circumferential length change of knee (cm) (post-OP day 2)	2.8 ± 0.9	3.1 ± 0.6
Extensive ecchymosis status (post-OP 2 weeks)	2	1
Subcutaneous hematoma (post-OP 2 weeks)	2	3
Wound dehiscence (post-OP 2 weeks)	1	0
Deep-vein thrombosis (post-OP 2 weeks)	0	1 (suspected)
Wound infection (post-OP 6 weeks)	0	0
Deep periprosthetic joint infection (post-OP 6 weeks)	0	0
Reoperation due to any reasons (post-OP 2 years)	0	0
Readmission for general complications (post-OP 2 years)	0	0

**Table 4:** Effect size measured by Cohen’s d.

	Cohen’s d
Estimated total blood loss	0.032382
Calculated blood loss	0.094433

## Data Availability

The data presented in this study are available upon request from the corresponding author. The data are not publicly available due to privacy.

## Supplementary Materials

The following supporting information can be downloaded at: https://www.mdpi.com/article/10.3390/medicina59122078/s1, Table S1: CONSORT 2010 checklist; Table S2: Estimated total blood loss formula of information to include when reporting a randomized trial [19,20].
