# Supplementary material for: Topical Fibrin Sealant (Tisseel@) Does Not Provide a Synergic Blood-Conservation Effect with Tranexamic Acid in Total Knee Arthroplasty—A Prospective Randomized Controlled Trial
Source: Medicina (Kaunas). 2023 Nov 26;59(12):2078. doi: 10.3390/medicina59122078 (PMC10744547; doi:10.3390/medicina59122078)
Supplement: Supplementary file 1 [file medicina-59-02078-s001.zip › Table S2. Estimated total blood loss formula.docx]

Table S2. Estimated total blood loss formula

| Total blood loss was calculated based on Gross formula [19] |
| --- |
| Total RBC volume loss (L) = patient’s blood volume (PBV)(L) × (Hct_pre_− Hct_post_)  Total blood loss (L) = (Total RBC volume loss (L) + (Number of units transfused × 0.285)) ÷ Hct_ave_  Hct_pre_ = the initial preoperative hematocrit level  Hct_post_ = the lowest postoperative hematocrit level during hospitalization or the lowest postoperative hematocrit prior to blood transfusion  Hct_ave_ = the average of the Hct_pre_ and Hct_post_  On average, one unit of packed red blood cells transfusion contains 0.285L of RBC volume |
| **PBV was calculated according to the formula of Nadler et al.** **[20]** |
| PBV (L) = k1 × height (m) ^3^ + k2 × weight (kg) + k3  For male patients: k1 = 0.3669, k2 = 0.03219, and k3 = 0.6041  For female patients: k1 = 0.3561, k2 = 0.03308, and k3 = 0.1833 |
